# Supplementary material for: MetaRibo-Seq measures translation in microbiomes
Source: Nat Commun. 2020 Jun 29;11:3268. doi: 10.1038/s41467-020-17081-z (PMC7324362; doi:10.1038/s41467-020-17081-z)
Supplement: Supplementary file 10 — Supplementary Data 7 [file 41467_2020_17081_MOESM10_ESM.zip › File2/Confidence_High_Taxonomy/308814_out.krona.html]

Javascript must be enabled to view this page.

members
magnitude
magnitudeUnassigned
count
unassigned
taxon
rank

308814\_out

3

superkingdom
3
2

phylum
3
976

3
200643
class

3
171549
order

815
3
family

genus

SRS043001\_contig\_number\_5279
3
816
1


SRS016018\_contig\_number\_16781SRS046717\_contig\_number\_5808
species
457387
2
